# The Principles of Medical Organisation

**Published:** 1903-08

**Authors:** 


*EDITOR.*

WILLIAM WARREN POTTER, M. D.

All communications, whether of a literary or business nature, books for review and exchanges should be addressed to the editor: 284 FRANKLIN STREET, BUFFALO, N. Y.

VOL. XLIII.—LIX. AUGUST, 1903. No. 1.

### The Principles of Medical Organisation

MEDICAL organisation is the paramount topic of the day. In view of this fact possibly it will not be a waste of time to review some of the principles involved in the formation, or the evolution of medical societies,—in an inquiry into the reasons for and methods of this movement.

We would observe that, following the law of developing life, medical societies must evolve in the direction of more in number and greater in individuality; this is innate and inevitable. As the primitive forms of life show several functions performed by a single structure, so the primitive medical societies, those of fifty years ago, were at once administrative, executive, scientific and social. From increase in numbers of physicians, from experience and augmented responsibility, from the widening and deeping of the field of medicine, from the rapid growth of the various departments of state medicine, from the rapid rise of specialties in medicine comes the inevitable demand for more, newer, more flexible, more convenient, more democratic, more highly specialised societies. This demand is but the growth of a living profession, the imperious claim of a higher life for opportunity to speak, to demonstrate, to perform, even to live. It is evidence of progress, and this interesting situation needs to be understood and encouraged.

However, we may offer a warning against possible waste of effort, disappointment, and arrest of progress, from failure to see clearly that medical societies, and particularly more highly specialised societies, must have precise rather than general functions. When societies are multiplying with rapidity there is danger that all may not see clearly what work belongs to each; some confusion is liable, some jostling possible, until each finds its precise place and its individual work. Of the more serious societies of New York,—state, district, and county,—it is to be observed that they are legal corporations, having the general powers and privileges of corporations and the special powers and privileges for which they were created. All of these powers, general and special, may be classed as executive, administrative, scientific, social and economical.

It is also to be noted that medical societies which are executive and administrative, must be constructed and conducted upon different lines from those which are economic and social in purpose. For instance, the early societies,—those constituted by the medical law of 1806, and its amendments,—were in the highest degree inclusive. They were simple in their purposes,—namely, "the diffusion of true science and particularly the knowledge of the healing art." Membership in the county societies was not optional, but obligatory. The annual due was fixed by law and was low; ability to make by-laws was limited by the principle that membership was not optional, but obligatory; therefore, by-laws could not include any topic unless the same was plainly germane to the purposes of the organisation.

This, it will be seen is widely different from the plan of the more recent societies, that is, those of 1900 known as the associations,—state, district, and county. Here, coupled with general medical purposes are business ambitions,—life insurance, publication enterprises, defense from malpractice suits, and other laudable pursuits. They are not, however, of such general nature as to demand the support of all medical men. We, therefore, find that in the associations membership is not obligatory but optional, and power to fix annual dues is greater, while dues are many times higher, and by-laws may include many topics forbidden to the general societies, upon the common sense principle that as membership is optional no harm can result from such by-laws, or, at least, the aggrieved member has always the protective remedy of resignation.

Again, this principle of inclusion or exclusion is found to vary largely from the changing needs of the scientific life of medical societies. When the societies of 1806 were formed, specialism in medicine was unknown and special societies were not required; hence, for scientific purposes societies could be inclusive as to membership. For like reasons now, all men do not have time for, or feel equal interest in, all departments of medical knowledge and we, therefore, must have societies which are exclusive as to their membership,—and this without necessary rivalry or competition. Hence, the special societies.

There has been for years rivalry and competition in this state between our several state societies, and for reasons not entirely to our credit. The American Medical Association, in 1851, adopted a report which substantially set forth that for a physician to believe in homeopathy was a misdemeanor, and was a just ground for expulsion from his county society, the majority of the profession having forgotten that the individual physician has a complete right to his opinion, even when that opinion differs from their own. As a result of this action the unity of the medical profession which had existed for half a century was broken in 1857, by the grant of a charter to the homeopathic physicians of New York for societies,—state and county. And unity was still further broken in 1865, by a like grant to the eclectic physicians. These grants were in all essentials like that of 1806, and the societies so formed were rivals.

Should misfortune prevail, this confusion and rivalry may possibly be still further increased by charter grants to some new sect. The future seems secure, however, in this respect, that before recognition the new sect will have to show willingness to meet the standards for state license in the fundamentals of medical knowledge. This will keep out the osteopaths and the Eddyites. Since 1882, the rivalry between the several medical societies of the state has been more apparent than real, the alliance then undertaken in the campaign for state license being a virtual concession and recognition of the legal and professional rights of the homeopathics and eclectics, and a virtual ending of any further possible rivalry, even if the disparity in age and in number of adherents had not already made such rivalry difficult, if not impossible.

Rivalry and competition between the state society of 1806, and the state association of 1900, are impossible, because the members of the association do not constitute a new sect in medicine. The future of the societies and associations of the State of New York will be determined by the services they will severally render to the multiplying and expanding interests of the medical profession; those that serve the most are the more likely to survive. In the meantime, we may confidently expect that a single society of delegates and permanent members, like the Medical Society of the State of New York, and a single county society in each county, simple, democratic, and inclusive in character, soon will come to exercise the entire executive and administrative concerns of the medical profession of the state. When this occurs, the homeopathic and eclectic societies,—state and county,—need not cease to exist, but may with the associations and the special societies continue to do scientific, social, or economic work, which voluntary and exclusive societies are peculiarly fitted to perform. The profession of the State of New York is about ready for such an organisation, and when the times are fully ripe, it will be organised.

